# New Onset of Diabetes Mellitus and Associated Factors among COVID-19 Patients in COVID-19 Care Centers, Addis Ababa, Ethiopia 2022

**DOI:** 10.1155/2022/9652940

**Published:** 2022-11-12

**Authors:** Asaminew Habtamu Sane, Migbar Sibhat Mekonnen, Melsew Getnet Tsegaw, Wuletaw Chane Zewde, Edmialem Getahun Mesfin, Hailu Asmare Beyene, Taye Mezgebu Ashine, Kasie Gebeyehu Tiruneh, Melkie Ambaw Mengistie

**Affiliations:** ^1^School of Nursing, Institute of Health Science, Jimma University, Jimma, Ethiopia; ^2^Department of Nursing, College of Health Science and Medicine, Dilla University, Dilla, Ethiopia; ^3^Millennium COVID-19 Care Center, Saint Paul's Hospital Millennium Medical College, Addis Ababa, Ethiopia; ^4^School of Nursing, College of Health Science and Medicine, Wolaita Sodo University, Sodo, Ethiopia; ^5^Schools of Nursing, College of Health Science and Medicine, Wachemo University, Hosaena, Ethiopia; ^6^Department of Nursing, Arba Minch College of Health Sciences, Arba Minch, Ethiopia

## Abstract

**Introduction:**

New onset of diabetes mellitus was noted as the commonest comorbidity in the COVID-19 pandemic, which contributed to a worse prognosis. Existing evidence showed that new-onset diabetes is associated with increased mortality compared to nondiabetic and known diabetic patients in the COVID-19 era. SARS-CoV-2 virus can worsen existing diabetes; at the same time, it can trigger new-onset diabetes that eventually worsens patient outcomes. Thus, this study is aimed at determining the prevalence and factors associated with new onset of diabetes mellitus among COVID-19 patients.

**Methods:**

Institution-based retrospective cross-sectional study design was conducted by reviewing 244 patient's records in the Addis Ababa COVID-19 care center. Descriptive statistics and binary logistic regression were used. During bivariate analysis, variables with *p* ≤ 0.25 were transferred into multivariate analysis. Adjusted odds ratios to determine the strength and presence of the association with a 95% confidence interval and *p* value ≤ 0.05 were considered, respectively.

**Results:**

The mean age of the study participants was 53.2 years with (SD = 13.35). The study findings showed that 31.1% (CI: 25.4-37.4) of COVID-19 patients had new onset of diabetes mellitus; of those, 11.8% had type 1 and 88.2% had type 2 diabetes. Being male (aOR = 2.9; 95% CI: 1.2, 7.1), family history of hypertension (aOR = 3.7; 95% CI: 1.3, 10.5), obesity (aOR = 3.1; 95% CI: 1.01, 8.9), having pulmonary embolism (aOR = 0.2; 95% CI: 0.06, 0.04), and hyperkalemia (aOR = 9.3; 95% CI: 1.8, 47.3) showed statistically significant association with new onset of diabetes mellitus.

**Conclusion:**

A significant proportion of COVID-19 patients had been diagnosed with new onset of diabetes mellitus, and new-onset type 2 diabetes mellitus is the most common diabetes mellitus type. Being male, obesity, having a pulmonary embolism, family history of hypertension, and hyperkalemia were independently associated with new onset of diabetes mellitus among COVID-19 patients. Therefore, focused interventions need to be strengthened towards the identified factors.

## 1. Introduction

Diabetes mellitus (DM) is a metabolic disorder resulting from a defect in the secretion or operation of insulin [1]. In December 2019, in Wuhan (China), the first cases of severe pneumonia of unknown origin were reported [2]. The causative organism has been identified as the severe acute respiratory syndrome coronavirus 2 (SARS-CoV-2) and has been identified as a new enveloped RNA betacoronavirus [3]. Globally, as of 2 March 2022, there have been 439,421,085 confirmed cases of COVID-19, including 5,987,035 deaths, reported to WHO [4].

Several studies have shown that new-onset diabetes is associated with COVID-19. The phenomenon of new-onset diabetes following admission to the hospital has also occurred with other viral infections and acute illnesses. The precise mechanisms for new-onset diabetes in people with COVID-19 are not known, but it is likely that several complexes and interrelated processes were involved, including previously undiagnosed diabetes, stress hyperglycemia, steroid-induced hyperglycemia, and direct or indirect effects of severe acute respiratory syndrome coronavirus 2 (SARS-CoV-2) on the *β*-cell [5]. In COVID-19 patients without or in conjunction with other comorbidities, new-onset diabetes has been associated with adverse clinical outcomes and high mortality [6–8].

Several studies have indicated that patients with new-onset diabetes are more likely to die, be admitted to the intensive care unit, and require intermittent mandatory ventilation assistance than those with healthy glucose levels and existing diabetes. Furthermore, COVID-19 patients with new-onset diabetes were found to have more severe complications such as ARDS, acute kidney injury, shock, hypoalbuminemia, and serious COVID-19 complications compared to those who did not develop ARDS or had hyperglycemia or normoglycemia [9–11]. Some of the existing findings showed that the new onset of diabetes mellitus is the major cause of morbidity and mortality, which leads to a large health and financial burden worldwide [12].

New-onset diabetes after COVID-19 is an emerging public health problem of increasing importance as the pandemic continues. Findings from several available studies suggest that COVID-19 indirectly poses a risk of new-onset diabetes in COVID-19 patients at many levels; in some countries, this may impede access to adequate health services. A disrupted supply of medicines, technologies, and care to people with DM may lead to poor glycemic control, leading to more complications [13, 14]. It also delayed an appropriate response to emergencies, as emergency department visits were reported to be significantly lower, largely because of concerns about infection [15]. In addition, the pandemic has been accused of exacerbating adaptive psychological difficulties in people with diabetes [16]. Studies conducted abroad show that patients with new-onset DM have an increased risk of death compared with patients with known diabetes and those without diabetes [12]. In addition, a large number of COVID-19 patients with new-onset hyperglycemia were identified as having chronic glucose intolerance and progressing to chronic DM after recovery from COVID-19 infection [17, 18].

At our COVID-19 management center in Ethiopia, we encountered new-onset diabetes mellitus (DM), one of the most common comorbidities and risk factors for adverse outcomes as demonstrated in our experience. Still, we could not find any publicly available data on new-onset diabetes in patients with COVID-19. This work is aimed at determining the extent, type, and possible associated factors of new-onset diabetes in COVID-19 patients.

## 2. Methods and Materials

### 2.1. Study Design and Population

An institution-based cross-sectional study was conducted among 244 confirmed COVID-19 patients who were admitted to the Addis Ababa COVID-19 care center from September 2020 to September 2021. An overall 6210 COVID-19 cases were admitted to the center, 765 of those who had either known or new onset of diabetes mellitus. All confirmed COVID-19 patients with the help of reverse transcriptase-polymerase chain reaction (RT-PCR) diagnostic methods were the source population, and those diabetic patients were the study population. Charts without documented HbA1C and those aged less than 18 years old were excluded.

### 2.2. Sample Size Determination and Sampling Technique

After taking into account the 95 percent confidence level, 19.7 percent proportion of new cases of diabetes mellitus [12], and 5 percent degree of freedom, the required sample size was calculated using the single population proportion formula using the StatCalc program of Epi Info version 7.2.5 statistical software created by the CDC in Atlanta, Georgia (US). Consequently, 244 people were included in the sample. Following that, a methodical random sampling process was used to choose the samples. When the study population was divided by the sample size, the sampling interval (*K*) was computed and three was the result. As a result, every third chart was examined after the lottery approach had been used to choose the first instance.

### 2.3. Operational Definitions

According to the American Diabetes Association, new-onset DM refers to diabetes mellitus that developed after COVID-19 infection, which was declared by fasting plasma glucose (FPG > 126 mg/dl) or random blood glucose (RBS > 200 mg/dl) and HbA1C < 6.5% in patients with no preceding history of DM. Known DM is defined as diabetes that was already diagnosed before COVID-19 infection. Newly diagnosed DM stands for diabetes that occurred before COVID-19 infection but left undiagnosed until admission to the COVID-19 center. The diagnosis of newly diagnosed DM was confirmed using FPG > 126 mg/dl or RBG > 200 mg/dl and HbA1C > 6.5% [16]. Hemoglobin A1C, it is a simple blood test that measures average blood sugar levels over the past 3 months [17]. Overweight can be defined as a BMI of 25 and more, and obesity as an index of 30 and more [18].

### 2.4. Data Collection Tools and Quality Assure

Data was collected using a structured and pretested extraction checklist, which was designed from patient registration follow-up, and based on previous literature [7, 12, 19–21]. It includes sociodemographic characteristics, clinical and laboratory investigations, comorbidities, diabetes mellitus types, and management-related variables. Experts in the field initially validated the tool. Furthermore, the reliability check was performed using Cronbach's alpha (*α* = 0.86).Training was given on the data collection tools for four data collectors and one supervisor, who was an MSc holder in nursing.

### 2.5. Data Processing and Analysis

The data were entered into Epi data version 4.4.2.2 and exported to SPSS version 25 for analysis after already being checked for completeness. Data was described using frequencies, percentages, and numerical measures. Binary logistic regression was applied to conduct inferential statistical analysis. Bivariate analysis was done to determine the association of each independent variable with outcome variable (new-onset DM), and variables with a *p* value of < 0.25 were a candidate for a multivariable model. Multivariable analysis was run for confounder adjustment after conducting a model fitness test using the Hosmer*–*Lemeshow test. Then, an adjusted odds ratio was used to determine the association and strength with a 95% confidence interval, and statistical significance was declared at a *p* value of 0.05.

### 2.6. Ethical Declaration

The Saint Paul's Hospital Millennium Medical College institutional review board (IRB) approved the study. Afterward, an official cooperation letter was obtained from the research directorate to the clinical director of the Millennium COVID-19 Care Center; then, ethical approval code PM23/799 was given for us to study. Data collection was conducted after obtaining permission from the clinical director and record room officers. Because of the retrospective nature of this study, study participants and family member consent was waived. The study was carried out in a manner consistent with the ethical principles of the Declaration of Helsinki. Confidentiality was maintained at all levels of the study through anonymous data collection.

## 3. Results

### 3.1. Sociodemographic Characteristics

An overall 244 samples with confirmed COVID-19 and diabetes mellitus were incorporated with a response rate of 100%. The study findings showed that 153 subjects (62.7%) were male and around 54% had a family history of DM. The mean age of the study subjects were found to be 53.2 years (SD = 13.35) with a minimum of 22 and a maximum of 88 years. Greater than 31% [12] of patients with new-onset DM were above the age of 65 years, whereas only 13% [20] of those without new-onset DM were in the same age group (Table 1).

### 3.2. Description of Baseline Laboratory Investigations

Regarding the baseline laboratory investigation findings, 182 (74.6%) patients had HbA1C of ≥6.5 percentage, and 68.4% of them had a random blood glucose level of greater than 200 mg/dl. Twenty-five (10.2%) patients had a creatinine clearance of 1.2 mg/dl. The mean cholesterol level among the study participants was 257.7 mg/dl (SD = 76.99). Furthermore, 76.6% of the study subjects had a cholesterol level of 200 mg/dl or more, of which 29.9% had new-onset DM. On the other hand, 36.1% and 28.3% of patients were hyponatremic (Na^+^ < 135 mmol/l) and hypokalemic (K^+^ < 3.5 mmol/l) at admission, respectively. Conversely, 7.4% of them had hyperkalemia (K^+^ > 5.2 mmol/l) at baseline. More than three-fourths (77.1%) of the study subjects were hypoalbuminemia (<3.4 mg/dl) with an average albumin level of 2.9 mg/dl (SD = 1.23) (Table 2).

### 3.3. Description of Presenting Symptoms

The study results also revealed that the most frequently reported COVID-19 symptoms were cough, chest pain, dyspnea, sore throat, and fever, which were recorded in 82.8%, 79.9%, 78.3%, 51.2%, and 49.6% of the patients, respectively (Table 3).

### 3.4. Description of Comorbidities among Study Participants

One-hundred and ninety-seven patients had coexisting medical illnesses, of which 168 (85.3%) had two or more comorbidities. Hypertension was the commonest comorbidity among others that had been recognized among 68.4% of the study participants, followed by pulmonary embolism (50.8%), asthma (41%), and COPD (15.2%) (Table 4).

### 3.5. Prevalence of New-Onset DM

Based on the study finding, 31.1% (95% CI: 25.4, 37.4) of COVID-19 patients had new onset of diabetes mellitus (DM), and of those, 88.2% had type 2 DM. Among the 244 patients included in the study, 83.6% were diagnosed with type 2 DM and the rest 16.4% had type 1 DM.

### 3.6. Factors Associated with New-Onset DM

In the bivariate analysis, variables such as sex, family history of diabetes mellitus, history of hypertension, pulmonary embolism (PE), body mass index, anticoagulant, hyperkalemia, severity of illness, immune-suppressive drugs, and hyponatremia were found to be candidate variable for multivariable analysis with a *p* value of less than 0.25. Then, multivariable analysis was run by including these variables for confounder adjustment after performing model fitness and other assumption tests. Finally, male sex, higher body mass index, having a family history of hypertension, pulmonary embolism, and hyperkalemia at baseline showed a statistically significant association with the presence of new onset of diabetes mellitus among COVID-19 patients at a 95% confidence level.

The study findings revealed that male patients were 2.9 times more likely to develop new onset of diabetes mellitus [(aOR = 2.9; 95% CI: 1.2, 7.1), *p* = 0.018] than female patients. The odds of new-onset DM were 9.3 times higher among hyperkalemic patients compared to patients with normal potassium levels at admission [(aOR = 9.3; 95% CI: 1.8, 47.3), *p* = 0.007]. Likewise, patients with a family history of hypertension were 3.7 times [(aOR = 3.7; 95% CI: 1.3, 10.5), *p* = 0.012)] at an increased risk of experiencing new-onset DM compared to their counterparts. Additionally, the odds of new-onset DM were tripled among obese patients (BMI ≥ 30) compared with those with normal body weight (BMI = 18.5‐25) [(aOR = 3.1; 95% CI: 1.01, 8.90), *p* = 0.048)]. Lastly, the presence of pulmonary embolism was found to have a protective effect on new-onset DM [(aOR = 0.15; 95% CI: 0.06, 0.40), *p* = <0.001]. Patients with pulmonary embolism (PE) were 85% less likely to have new-onset DM compared to those without PE (Table 5).

## 4. Discussion

The COVID-19 outbreak is continuing to spread rapidly around the world, and it causes a novel pathophysiological change in glucose homeostasis (a combination of severe insulin resistance and insulin insufficiency) [13, 22]. New-onset diabetes after COVID-19 is an emerging public health problem of increasing importance as the pandemic continues. This cross-sectional study is aimed at determining the extent of new-onset DM in patients with COVID-19 and to identify factors associated with it.

This study revealed that 31.1% (95% CI: 25.4-37.4) of patients had COVID-19-associated new-onset DM. This finding is in line with a multicenter retrospective study that reported 29.1% of new diabetics among COVID-19 patients with no previous diagnosis of DM [2]. Conversely, other reports conducted in Zagazig 44519, Egypt (13.5%) and a systematic review and meta-analysis (19.7%) reported a lower prevalence of new-onset DM compared to the current study result [12, 21]. This discrepancy might be due to the difference in the prevalence of DM in the general population of Ethiopia (6.5%) [23] and other countries abroad (15.6% in Egypt and 10.2% in the USA) [24, 25]. This means that the higher prevalence of noncommunicable diseases such as DM in the developed world, where previous studies were conducted, could decrease the number of new-onset DM during the COVID-19 pandemic. On the other hand, the pooled prevalence of new-onset DM reported in the systematic review is mainly considered in studies that were published in the developed world. Hence, a large amount of diabetic patients with COVID-19 infection in these countries will reduce the number of new COVID-19-associated diabetic cases.

Our study also revealed that the mean age of the study participants was 53.2 and the new-onset diabetic patients had significantly older ages than not new-onset diabetes patients, 56.8 vs. 52.65, respectively. This was in agreement with a study done in Zagazig 44519, Egypt, that reported the newly diagnosed diabetic patients had significantly older age 57.7, and it was also in agreement with Li et al., who reported that COVID-19 patients with newly onset DM and hyperglycemia were slightly older and obese [21, 22]. A possible reason for the link between older age and new-onset DM was that in our findings, type two diabetes was the most common; therefore, it most commonly occurs in older age (individuals aged 35–70 years).

HbA1C was performed for all newly diagnosed diabetic patients to differentiate between new-onset and preexisting diabetes mellitus (DM) accordingly; our current study showed that new-onset type 1 DM developed in nine (22.5%) patients and 67(32.8%) patients had new-onset type 2 diabetes mellitus (DM). This finding was consistent with a study done in Egypt that revealed out of 77 newly onset diabetic patients, new-onset type 1 DM developed in 7 patients (1.2%), and 58 patients (10.2%) had new-onset type 2 diabetes mellitus(DM) [21]. However, in some studies, HbA1C was not performed for all participants, so it was not possible to differentiate between new-onset and previously undiagnosed diabetes [2, 26].

The study findings revealed that male patients were 2.9 times more likely to develop new onset of diabetes mellitus [(aOR = 2.9; 95% CI: 1.2, 7.1), *p* = 0.018] than female patients. This association was supported by a study conducted in South Korea on 5,307 COVID-19 patients in that males have a 61.6% higher chance of developing DM compared to females [22]. The scientific justification for this association could be that men are more prone to develop diabetes because of biological factors. In addition, men can acquire diabetes with less weight gain compared to females [27].

The odds of new-onset DM were 9.3 times higher among hyperkalemic patients compared to patients with normal potassium levels at admission [(aOR = 9.3; 95% CI: 1.8, 47.3), *p* = 0.007]. This association is not evidenced in recently reported studies worldwide. The rationale could be due to the physiologic link between diabetes and elevated potassium levels. In the general population, diabetes is associated with a higher incidence of hyperkalemia [28, 29]. Redistribution of potassium from the intracellular to the extracellular compartment can induce hyperkalemia with no net total body potassium increment. Shift hyperkalemia in DM can result due to acidosis, insulin deficiency, hypertonicity, cell lysis (rhabdomyolysis), and drugs (e.g., betablockers). For every 0.1 falls in pH, potassium increases by approximately 0.4 mmol/l in patients with metabolic acidosis [28, 29].

Likewise, patients with a family history of hypertension were 3.7 times [(aOR = 3.7; 95% CI: 1.3, 10.5), *p* = 0.012)] at an increased risk of experiencing new-onset DM compared to their counterparts. This finding is consistent with the findings of previous studies conducted in Egypt by Farag et al. [21] and a systematic review and meta-analysis [30]. Possibly, this could be explained by the effect of genetics that plays a key role in the development of multiple diseases including diabetes and hypertension [31, 32]. Hypertension is the commonest comorbidity among diabetic patients. High blood pressure can cause insulin resistance, even when kept within the high normal range, which is associated with an increased risk of diabetes. Having a family history of hypertension is reported to be associated with an increased risk of diabetes and speeds up diabetes occurrence. A retrospective study conducted on 1299 patients revealed that patients with a family history of hypertension develop diabetes mellitus two years earlier than those without a family history [32].

Obesity was the other factor that had a statistically significant connection. In comparison to patients with a normal body weight (BMI = 18.5‐25), obese patients (BMI ≥ 30) had a threefold increased risk of developing new-onset diabetes [(aOR = 3.1; 95 percent CI: 1.01, 8.90), *p* = 0.048]. This result is consistent with reports from studies carried out in Egypt, South Korea, China, and Indonesia, as well as a systematic review [6, 9, 21, 33]. The explanation might be that having a higher BMI increases the likelihood of developing new-onset diabetes by inducing insulin resistance and hastening the course of the disease [34–38].

Pulmonary embolism was found to have protective effect on new-onset DM [(aOR = 0.15; 95% CI: 0.06, 0.40), *p* = <0.001]. Patients with pulmonary embolism (PE) were 85% less likely to have new-onset DM compared to those without PE. No statistically significant association was reported in earlier studies done to assess the association between PE and new-onset DM [39]. Hyperglycemia causes activation of coagulation and hypofibrinolysis, leading to embolism, conversely, venous thromboembolism (VTE), which is often accompanied by acute hyperglycemia or stress hyperglycemia through different mechanisms [40–42]. First, elevated glucose levels during embolization can result from inflammatory and counterregulatory hormone action in response to the physical stress by the VTE event itself. Second, undiagnosed impaired glucose tolerance may be present in a proportion of patients before the VTE itself and may, therefore, have contributed to the development of thrombosis. An increase in the incidence rate of DM is evidenced in patients after a diagnosis of VTE [39]. This evidence is applicable for both new-onset and known diabetic patients. However, patients with known diabetes mellitus are at an increased risk of PTE, which increases the chance of a frequent diagnosis of PE compared with those with new-onset diabetes.

The possibility of including stress hyperglycemia as new-onset DM may overestimate its prevalence. Due to the retrospective study, the effect of family history of diabetes, dietary history, and patients' lifestyle factors on new-onset DM among COVID-19 patients, it might also be difficult to draw strong inferences since the available data were not primarily designed for research purposes.

## 5. Conclusion

According this study, there was a significant frequency of new onset of diabetes, the majority of which was type 2. An increased risk of new onset of diabetes mellitus was linked to being male, having a history of prior hypertension, being obese (BMI ≥ 30), and having high blood potassium levels at admission. Furthermore, it was discovered that pulmonary embolism was protected against the onset of new onset of diabetes. In individuals with COVID-19, ceasing these well-established factors may assist patient's control on the effects of new-onset diabetes.

## Figures and Tables

**Table 1:** Sociodemographic information about the study participants at COVID-19 care center in Addis Ababa, Ethiopia, in 2022 (*N* = 244).

Parameter	Category	DM		Total
		New-onset (%)	Not new-onset (%)	
Sex	Male	63 (41.2%)	90 (58.8%)	153 (62.7%)
	Female	13 (31.7%)	78 (85.7%)	91 (37.3%)
Age	<65	52 (68.5%%)	146 (87.1%)	198 (81.1%)
	≥65	24 (31.5%)	22 (13.1%)	46 (18.8%)
Residence	Addis Ababa	63 (35.2%)	116 (64.8%)	179 (73.4%)
	Regional	13 (20.0%)	52 (80.8%)	65 (26.6%)
Family history of DM	Yes	41 (36.7%)	72 (63.7%)	113 (46.3%)
	No	20 (26.7%)	55 (73.3%)	75 (30.7%)
	Unknown	15 (26.8%)	41 (73.2%)	56 (23%)
History of hypertension	Yes	47 (35.1%)	88 (65.6%)	134 (54.9%)
	No	29 (26.4%)	81 (73.4%)	110 (45.1%)
Severity of illness	Mild	3 (25%)	9 (75%)	12 (4.9%)
	Moderate	26 (24.3%)	81 (75.7)	107 (43.8%)
	Severe	32 (25.6%)	93 (75%)	125 (51.2%)
Types of DM	Type one DM	9 (11.8%)	31 (77.5%)	40 (16.4%)
	Type two DM	67 (88.2%)	137 (67.2%)	204 (83.6%)

Key: DM: diabetes mellitus.

**Table 2:** The result of a laboratory investigation of the study participants at the COVID-19 care center in Addis Ababa, Ethiopia, in 2022 (*N* = 244).

Parameter	Category	Diabetes mellitus		Total
		New-onset	Not new-onset	
Potassium	≤3.5 mEq/l	18 (26.1%0	51 (73.9%)	69 (28.3%)
	3.51-5 mEq/l	47 (29.9%)	110 (70.1%)	157 (64.3%)
	≥5 mEq/l	11 (61.1%)	7 (38.9%)	18 (7.3%)

BUN	<21 mg/dl	0	13 (100%)	13 (5.3%)
	≥21 mg/dl	76 (32.9%)	155 (67.1%)	231 (94.6%)

Sodium	≤35 mEq/l	22 (25.0%0	66 (75%)	88 (36.1%)
	>35 mEq/l	54 (34.6%)	102 (65.5%)	156 (63.9%)

Creatinine	≤1.2 mg/dl	70 (32.0%)	149 (68%)	219 (89.7%)
	≥1.21 mg/dl	6 (24.0%)	19 (76.0%	25 (10.2%)

Cholesterol (hyperlipidemia)	≤200 mg/dl	20 (35.1%)	37 (64.9%)	57 (23.3%)
	>200 mg/dl	56 (29.9%)	131 (70.1%)	187 (76.6%)

Albumin	≤3.4 mg/dl	47 (25%)	141 (75%)	188 (77%)
	>3.4 mg/dl	14 (25%)	42 (75%)	56 (22.9%)

Key: BUN: blood urea nitrogen.

**Table 3:** Clinical characteristics of the study participants at the time of their admission to the COVID-19 care facility in Addis Ababa, Ethiopia, in 2022 (*N* = 244).

Parameter	Category	Diabetes mellitus		Total
		New-onset DM	Not new-onset DM	
Fever	Yes	31 (25.7%)	90 (74.3%)	121 (50%)
	No	45 (36.6%)	78 (63.4%)	123 (50.4%)
Sore throat	Yes	41 (32.8%)	84 (67.2%)	125 (51.2%)
	No	35 (29.42%)	84 (70.6%)	119 (48.8%)
Cough	Yes	63 (31.3%)	139 (68.8%)	202 (82.8%)
	No	13 (31%)	29 (69%)	42 (17.2%)
Polyuria	Yes	41 (30.1%)	95 (69.9%)	136 (55.7%)
	No	35 (32.4%)	73 (67.6%)	108 (44.3%)
Polydipsia	Yes	41 (28.7%)	102 (71.3%)	143 (58.6%)
	No	35 (34.7%)	66 (65.3%)	101 (41.4%)
Shortness of breath	Yes	54 (28.3%)	137 (71.7%)	191 (78.3%)
	No	22 (41.5%)	31 (58.5%)	53 (21.8%)
Chest pain	Yes	59 (30.3%)	136 (69.7%)	195 (79.9%)
	No	17 (34.7%)	32 (65.3%)	49 (20.1%)

**Table 4:** Summaries on comorbidities of research participants at COVID-19 care center, Addis Ababa, Ethiopia, 2022 (*N* = 244).

Parameter	Category	DM		Total
		New-onset DM	Not new-onset DM	
Comorbidity	Yes	63 (35.2%)	116 (64.8%)	179 (73.3%)
	No	13 (20%)	52 (80%)	65 (26.6%)
Hypertension	Yes	56 (33.5%)	111 (66.5%)	167 (68.4%)
	No	20 (26.0%)	57 (74.0%)	77 (31.5%)
COPD	Yes	9 (24.3%)	28 (75.7%)	37 (15.2%)
	No	672 (32.6%)	140 (67.6%)	207 (84.8%)
Pulmonary embolism (PE)	Yes	25 (20.2%)	99 (79.8%)	124 (50.8%)
	No	51 (42.8%)	69 (57.5%)	120 (49.2%)
Asthma	Yes	29 (29%)	71 (71%)	100 (40.9%)
	No	47 (32.6%)	97 (67.4%)	144 (59%)
Number of comorbidities	None	14 (29.8%)	33 (70.2%)	47 (19.3%)
	One	10 (34.5%)	19 (89.7%)	29 (11.9%)
	Two	21 (25.6%)	61 (74.4%)	82 (33.6%)
	Three and greater	31 (36.0%)	55 (64.4%)	86 (35.6%)
Patient outcome	Death	44 (57.8%)	91 (54.2%)	135 (44.6%)
	Recovered	32 (42.1%)	77 (45.8%)	109 (44.6%)

**Table 5:** Associations between specific variables and new onset of diabetes mellitus in COVID-19 care center, Addis Ababa, Ethiopia, 2022 (*N* = 244).

Parameter	Category	DM		cOR (95% CI)	aOR (95% CI)	Sig.
		New-onset	Not new-onset			
Sex	Male	63	90	4.2 (2.2, 8.2)	2.9 (1.21, 7.11)	0.018
	Female	13	78	1	1	
Family history of DM	Yes	41	72	0.6 (0.32, 1.32)	0.5 (0.17, 1.36)	0.167
	No	20	55	1.0 (0.52, 2,21)	2.2 (0.56, 8.31)	0.264
	Unknown	15	41	1	1	1
Pulmonary embolism (PE)	Yes	25	99	2.9 (1.72, 5.22)	0.2 (0.06, 0.40)	<0.001
	No	51	69	1	1	
Body mass index	18-<25	31	95	1	1	.488
	25-<30	16	59	0.78 (0.41, 1.45)	4.2 (1.51, 11.6)	
	≥30	11	29	1.4 (1.11, 5.42)	3.1 (1.01, 8.9)	0.048
Family history of hypertension	Yes	47(35.1%)	88(65.6%)	1.5 (1.01-3.62)	3.7 (1.31, 10.5)	0.012
	No	29(26.4%)	81(73.4%)	1	1	
Anticoagulant	Heparin	68	136	0.5 (0.21, 1.22)	0.4 (0.10, 1.62)	0.19
	Enoxaparin	8	32	1	1	
Potassium	Hypokalemia	18	51	1	1	0.192
	Normal	47	110	1.2 (.642, 2.3)	1.7 (.762, 3.78)	
	Hyperkalemia	11	7	4.5 (1.52, 13.2)	9.3 (1.82, 47.3)	0.007
The severity of the illness	Mild	3	9	1.12 (0.29-4.4)	0.7 (0.1, 5.22)	0.740
	Moderate	26	81	0.65 (0.4, 1.2)	1.9 (0.72, 5.51)	0.202
	Severe	32	93	1	1	
Immune suppressive drug	Prednisolone and corticosteroid	18	54	0.65 (0.6, 1.28)	2.3 (0.90, 5.81)	0.082
	Dexamethasone	58	114	1	1	
Sodium	≤35	22	66	0.63 (0.4, 1.2)	1.4 (0.7, 2.96)	0.341
	>35	54	102	1	12	

Key: OR: odds ratio; aOR: adjusted odds ratio; CI: confidence interval; PE: pulmonary embolism; BMI: body mass index; DM: diabetes mellitus.

## Data Availability

All data supporting the study is available on the reasonable request.

## Abbreviations

aOR: Adjusted odds ratio
CDC: Center of Disease Control
cOR: Crude odds ratio
CI: Confidence interval
COVID-19: Coronavirus disease 2019
DM: Diabetes mellitus
ED: Emergency department
ICU: Intensive care unit
LMICs: Low- and middle-income countries
M3C: Millennium COVID-19 Care Center
RT-PCR: Reverse transcriptase-polymerase chain reaction
SPHMMC: Saint Paul's Millennium Medical College
SPSS: Statistical Package for the Social Sciences
SARS-COV-2: Severe acute respiratory syndrome coronavirus
WHO: World Health Organization.
